# Recruitment in Clinical Versus Community-Based Sites for a Pilot Youth Diabetes Prevention Program, East Harlem, New York, 2011–2012

**DOI:** 10.5888/pcd13.150449

**Published:** 2016-01-28

**Authors:** Nita Vangeepuram, Kenya Townsend, Guedy Arniella, Crispin Goytia, Carol R. Horowitz

**Affiliations:** Author Affiliations: Kenya Townsend, Crispin Goytia, Carol R. Horowitz, Department of Population Health Science and Policy, Icahn School of Medicine at Mount Sinai, New York, New York; Guedy Arniella, Institute for Family Health, New York, New York.

## Abstract

**Introduction:**

Little is known about successful strategies for recruitment of youth for research. The objective of this study was to compare clinical sites with community sites in the recruitment of teenagers for a new youth diabetes prevention program in East Harlem, New York.

**Methods:**

We assessed diabetes risk for youth (aged 13–19 y) by measuring body mass index (BMI). We then screened overweight and obese youth for prediabetes using oral glucose tolerance testing, had them complete a health and lifestyle survey, and enrolled prediabetic youth into peer-led workshops. The recruitment strategies were 1) clinical referrals and 2) screenings at community sites. We compared the number of adolescents screened, the proportion eligible for testing, the proportion diagnosed with prediabetes, baseline characteristics, and the retention rates between those recruited in clinical and community sites.

**Results:**

In 3 months, we completed BMI screening for 156 adolescents from community sites and 30 from clinical sites. Overall, 47% were at risk for diabetes on the basis of BMI, and 63% returned for diabetes testing; 35% had prediabetes, and 1 teenager had diabetes. Clinical sites yielded higher rates of diabetes risk on the basis of BMI and higher rates of return for screening and diagnosed prediabetes. Although demographics and BMI did not vary by recruitment site, we found differences in behaviors, self-efficacy, body image, and social support. There were no differences by recruitment site in workshop enrollment or completion or return for follow-up.

**Conclusion:**

Both recruitment strategies were successful, and participants from both groups had high rates of undiagnosed prediabetes. Our approach allowed access to more adolescents and opportunities for education about diabetes in the community.

## Introduction

Type 2 diabetes has become increasingly prevalent among children and adolescents over the past few decades. Among adolescents aged 12 to 19, 16% have prediabetes, and rates increase with obesity prevalence (12% if normal weight, 18% if overweight, and 30% if obese) (1). Unless preventive measures are taken among prediabetic youth, type 2 diabetes in the United States will increase in this group by 49% by 2050, with the greatest increases among racial/ethnic minority youth (2). Therefore, there is a need to develop youth diabetes prevention programs and to recruit youth into studies to examine the effectiveness in reducing future diabetes risk, especially for youth from low-income, racial/ethnic minority communities, who are at highest risk for obesity, prediabetes, and diabetes (3).

Historically, it has been difficult to engage youth from high-risk communities in research (4). Many barriers to recruitment into healthy lifestyle intervention studies exist (5,6), and racial/ethnic minority youth from low socioeconomic backgrounds often face additional challenges (7,8).

Collaboration with community partners may be an effective strategy to recruit adults into diabetes prevention studies in high-risk communities. One study (9) found that a partner-led approach, in which community partners developed and managed the recruitment efforts at their sites, was the most effective recruitment strategy (accounting for 68% of participants enrolled vs 0% recruited through clinical sites) and the most efficient strategy (34% of those approached through partners enrolled vs 0% approached through clinical providers). However, no studies have compared the effectiveness and efficiency of community recruitment strategies with those of clinical recruitment strategies among youth.

This study examined how community, clinical, and academic partners worked together on study design and recruitment of participants into an adolescent-led diabetes prevention program, TEEN HEED. We describe recruitment procedures for the pilot intervention and compare recruitment at clinical and community sites.

## Methods

The study was conducted in East Harlem, also known as El Barrio, in the northeast corner of Manhattan. Its residents, of whom 55% are Latino and 33% are black, are predominantly low-income and have among the highest rates of obesity and diabetes mortality in New York City (10). The East Harlem Partnership for Diabetes Prevention is a community–academic partnership formed in 2005 to design and implement diabetes prevention strategies for the community. The Partnership developed Project HEED, Help Educate to Eliminate Diabetes, which used a community-based participatory research approach to design and test the effect of peer-led group workshops on weight among adults with prediabetes. In a randomized controlled trial, participants in the intervention group who were prediabetic had significant weight loss maintained at 1 year and a leveling of blood glucose levels, compared with controls (11).

In 2010, the Partnership’s Community Action Board expressed interest in expanding diabetes prevention efforts to young people. The Partnership formed a separate teen diabetes prevention action board that included clinicians, community leaders, and youth to maximize efforts to engage youth. Community, clinical, and academic partners modified Project HEED to create a developmentally and culturally appropriate youth diabetes prevention program, TEEN HEED. The goal of the pilot study, which took place from December 2011 through November 2012, was to screen at-risk adolescents for prediabetes and to enroll adolescents with prediabetes into an 8-week, peer-led, diabetes prevention workshop and conduct follow-up assessments postintervention. The institutional review board at the Icahn School of Medicine at Mount Sinai approved the study protocol.

The TEEN HEED Community Action Board (board) Recruitment Subcommittee developed communication, marketing, outreach, and recruitment strategies to help enroll teens in the study. Because the board included representatives from both clinical and community-based sites with a longstanding history of working with East Harlem youth, members decided to use 2 recruitment strategies: 1) screenings at collaborating community-based organizations with youth programs and 2) referrals from health care providers.

The board created a list of community-based organizations for recruitment, including sites with board representatives and sites where board members had close contacts. These sites were all youth-serving agencies that provide services such as after-school, recreational, leadership, and mentoring or tutoring programs. Board members helped develop overall community recruitment strategies and tailored these strategies for their sites, where they facilitated recruitment events. Youth leaders made the first outreach to teens about the study by distributing flyers and making announcements before recruitment events and introducing study staff during the events. Leaders scheduled events during times when other entertaining activities were occurring at the community sites so that more teens would be likely to be present. We engaged youth at these events using young, high-energy research staff and posters with messages about diabetes prevention: “Together we beat diabetes!” and “1 in 2: Don’t let it be you!” We divided the teens into 2 equal groups to demonstrate that approximately half of the teens present may be at risk for developing diabetes. We had a group discussion in which teens shared stories about people they know with diabetes and how living with diabetes affects their lives. We then gave details about the study, answered any questions, and assessed eligibility of interested adolescents. We assessed diabetes risk by measuring body mass index (BMI). When youth were identified as overweight or obese (BMI ≥85th percentile for age and sex) (12), we communicated this information in a private setting and provided information about the prediabetes screening and other study components. We then obtained parental consent and adolescent assent or adolescent consent (for participants >18 y). Board members (including teens) reviewed consent documents to ensure that they reflected all important details of the study and would be easily understood by parents and study participants.

For recruitment in clinical sites, we engaged pediatricians and family physicians in academic and community-based health centers. Again, the board helped develop an overall recruitment strategy that we tailored for sites on the basis of provider recommendations. In the 2 general pediatrics clinics (1 hospital-based clinic and 1 community-based clinic), primary care providers shared information about the study with patients whom they thought would be at risk for developing diabetes and interested in participating in the study. We then contacted parents of the patients and provided more details about the study and scheduled a time for eligibility assessment and consent. We used a similar strategy at a local hospital where a health educator shared study information with adolescents participating in a weight management program. We used a slightly different strategy at a local adolescent health center, where, as recommended by that center’s clinicians and outreach workers, research staff approached patients in the waiting room and provided a brief introduction to the study and then assessed eligibility and provided more detailed information about study requirements and consent for those who were eligible and interested.

We asked eligible participants (overweight and obese teens aged 13–19 who were not pregnant and were without known diabetes or prediabetes) recruited from both community and clinical sites to return for prediabetes screening after an overnight fast. We screened youth for prediabetes using oral glucose tolerance testing (13) and completed adiposity and blood pressure measurements and a health and lifestyle survey (most questions adapted from previously validated surveys used among adolescents) (14,15). We invited adolescents with prediabetes to participate in 8 weekly peer-led diabetes prevention workshops. Workshops covered behavioral skills, including goal setting (weekly action plans), self-monitoring, brainstorming, problem solving, contingency management, coping skills, and social support. Workshop topics included explanation of prediabetes and diabetes, label reading, healthy plate planning, portion control, finding affordable healthy foods, strategies to increase physical activity, and coping with eating triggers and social pressures. Participants returned for follow-up at 3 months to repeat all the assessments completed at baseline. Participants received incentives at each study visit (movie tickets or gift cards, suggested by the board as being popular but not coercive in this age group).

We collected data about the number of adolescents who completed BMI screening, who consented and returned for prediabetes testing, and who were diagnosed with prediabetes. The analytic team used simple descriptive statistics to compare the number of adolescents screened (measure of recruitment strategy effectiveness) and the proportion eligible for prediabetes testing and diagnosed with prediabetes (measure of recruitment strategy efficiency) between those recruited in clinical and community sites. The team next analyzed survey data for 55 adolescents (excluding 1 girl who was newly diagnosed with diabetes at baseline and therefore not eligible) using descriptive statistics and bivariate analyses (χ^2^ and *t* tests) to compare baseline characteristics of participants recruited in clinical versus community sites. Finally, we examined the number of participants with prediabetes from clinical and community sites who enrolled in workshops, completed workshops, and came for follow-up assessments. We completed all statistical analyses using SPSS Statistics for Windows, version 22.0 (IBM Corporation), with significance set at *P* < .05.

## Results

In 3 months, we prescreened 186 adolescents (84% from community sites and 16% from clinical sites) for study eligibility by assessing BMI (Table 1). Overall, nearly half were at risk for diabetes based on measured BMI. Of those who were eligible, approximately two-thirds returned for prediabetes testing, one-third of whom had prediabetes, and 1 had diabetes (and was excluded from further analysis). Comparing recruitment in clinical and community sites, we completed BMI screening with more teens in community sites but found higher rates of overweight and obesity and had higher rates of return for prediabetes testing among youth from clinical sites (Table 1). Clinical sites yielded a higher population of adolescents with previously undiagnosed prediabetes (nearly half) compared with community sites (a little more than one-quarter), although the difference was not significant (*P* = .10). Clinical referrals therefore provided a more efficient way to diagnose adolescents with prediabetes (10 of the 30 clinician-referred adolescents were diagnosed with prediabetes [33%] compared with only 9 of 156 adolescents from community sites [6%]).

**Table 1:** Recruitment in Clinical Versus Community Sites for a Pilot Youth Diabetes Prevention Program in East Harlem, New York, 2011–2012

Recruitment Site	No. Screened	High Diabetes Risk Based on Overweight/Obese BMI, No. (%)	Returned for Testing, No. (%)	Prediabetes Diagnosis, No. (%)
Total	186	88 (47)	55 (63)	19 (35)
Clinical	30	26 (87)	21 (81)	10 (48)
Community	156	62 (40)	34 (55)	9 (26)

Abbreviation: BMI, body mass index.

Survey data were available from all 55 eligible adolescents (62% female, 58% Hispanic, and 42% non-Hispanic black) who completed prediabetes testing. Demographic characteristics (sex, race/ethnicity, age, and level of parent education) and BMI of adolescents completing prediabetes testing did not differ by recruitment site. However, adolescents from community sites generally reported more healthful behaviors, higher physical activity self-efficacy, better body image, and higher levels of friend support for healthful behaviors than did adolescents from clinical sites (Table 2). Adolescents from clinical sites reported eating more meals with their families and higher levels of family support for healthful behaviors than did adolescents from community sites.

**Table 2:** Differences in Behaviors, Attitudes, and Social Support for Adolescents Recruited for a Diabetes Prevention Program, Clinical Versus Community Sites, East Harlem, New York, 2011–2012^a^

Variable	Community (n = 34)	Clinical (n = 21)	*P* Value^b^
Previously undiagnosed prediabetes	9 (27)	10 (48)	.10
Eat larger-than-recommended portion of cereal	16 (52)^c^	18 (86)	.01
Order large/extra-large portion of fast food	2 (6)	5 (24)	.05
Eat breakfast ≥4 days per week	22 (65)	8 (38)	.10
>4 Weekly hours of vigorous physical activity	13 (39)^d^	2 (10)	.03
Any time spent playing video games last week	13 (38)	14 (67)	.04
Perceive self as more active than others	13 (38)	4 (19)	.10
Physical activity self-efficacy score^e^ (range, 6–30), mean (SD)	21.8 (5)	19.0 (5)	.05
Self-esteem score^e^ (range, 6–24), mean (SD)	18.2 (3)	16.6 (3)	.08
Body satisfaction score^e^ (range, 10–60), mean (SD)	37.0 (9)	32.2 (8)	.08
Media internalization of body image score^f^ (range, 4–16), mean (SD)	7.6 (2)	9.5 (2)	.007
Level of friend support score^e^ (range, 5–20), mean (SD)	13.2 (2)	11.6 (1)	.006
Level of family support score^e^ (range, 5–20), mean (SD)	13.6 (2)	14.9 (2)	.05
Eat meals with family >4 times week	10/34 (29)	11/21 (52)	.06

Abbreviation: SD, standard deviation.

a Values expressed as no. (%), unless otherwise indicated.

b *P* values obtained using χ^2^ test for categorical variables and *t* test for continuous variables.

c Data available for 31 of 34 participants.

d Data available for 33 of 34 participants.

e Higher values indicate higher self-efficacy, self-esteem, body satisfaction, or level of support.

f A higher value correlates with more internalization of media images.

We found no differences by recruitment site in the proportions of teens with prediabetes enrolled in workshops (6 of 9 from community sites vs 7 of 10 from clinical sites, *P* = .90), completing workshops (5 of 6 from community sites vs 4 of 7 from clinical sites, *P* = .30), or returning for postintervention follow-up assessment (7 of 9 from community sites vs 9 of 10 from clinical sites, *P* = .50)

## Discussion

In this study, we formed a community–academic partnership that worked collaboratively to develop a pilot, community-based, peer-led intervention to prevent adolescent diabetes. We screened 186 adolescents for diabetes risk in less than 3 months. One in 3 overweight or obese adolescents had undiagnosed prediabetes, including nearly half of those recruited from clinical settings. The high prevalence of undiagnosed prediabetes was surprising, particularly because all adolescents reported having a regular health care provider, all were at high risk for prediabetes and diabetes, and, in the clinical setting, providers referred youth to our study because they were thought to be at high risk for diabetes. One potential explanation for this result is that hemoglobin A1c (a simple blood test routinely used by many pediatricians) is not validated as a way to diagnose prediabetes in youth (16), and most primary care providers do not perform oral glucose tolerance tests, even for patients who are at increased risk for diabetes on the basis of weight and family history of diabetes. Larger studies should be conducted to explore the best way to identify at-risk youth, to increase detection of prediabetes in simple, efficient ways outside the offices of pediatric endocrinologists, and to do so in clinical and nonclinical settings.

Adolescents are challenging to reach and engage in research (4–7). Recruitment for this study was particularly challenging because of the high burden of responsibility placed on participants. Once recruited and found to be at high risk for diabetes on the basis of BMI screening, participants were asked to return after an overnight fast for prediabetes testing, drink an oral glucose solution, stay for a half day of testing, and have repeated finger sticks. Despite this burden, we were able to successfully test nearly 60 adolescents, newly diagnose nearly 20 adolescents with prediabetes, engage youth in a workshop that most completed, and have adolescents return for follow-up testing.

Our success with recruiting and retaining study participants using strategies recommended by our community action board appears to align with findings from other studies. Traditional recruitment methods such as use of flyers or letters to potential participants, their parents, or their physicians may be helpful but are often insufficient to achieve recruitment goals (7). Instead, face-to-face recruitment (whether in community or clinical sites) may more successful than these conventional approaches (17,18). Having strong ties with collaborating community-based organizations and clinical sites (in our case via participation on our community advisory board, which developed the program’s conceptual model, study protocol, curriculum, evaluation methods, and recruitment strategies) fostered access to the target population, helped establish trust with potential study participants and their parents, and helped us develop engaging, youth-centered recruitment strategies (4,17,18). Enlisting young, enthusiastic recruiters who approached adolescents using appropriate language and allowing youth to make the initial decision to participate before discussing details with their parents may be an effective strategy (19). Monetary and material incentives that are attractive to participants (in our case, movie tickets and gift cards) and flexibility in scheduling study visits (including evening and weekend visits and home visits) may also have contributed to successful recruitment (4). Other strategies were building trust (as we often had to have multiple conversations with parents before consent was given), ensuring confidentiality, and being in continuous contact with the community sites, participants, and parents or guardians via telephone calls, text messages, emails, and letters to help with study retention (4,7,20).

To our knowledge, no previous studies have compared recruitment of youth in community versus clinical sites or identified differences in participant characteristics by recruitment site. In our study, community-based and clinical recruitment were both successful strategies for diagnosing at-risk adolescents with prediabetes. We completed BMI screening with fewer participants from clinical sites than from community sites (lower recruitment effectiveness), possibly because we relied on providers with busy clinical practices to introduce the study to their patients. Other studies also found that clinical referrals for participation in research may be challenging (9). However, despite the smaller proportion of participants recruited from clinical sites (less than 20%), about half of the youth we diagnosed with prediabetes were referred by clinicians. Clinical referrals thus provided a more efficient strategy to diagnose adolescents with prediabetes (higher recruitment efficiency). Although providers referred adolescents thought to be at-risk for diabetes, all adolescents attending programs in community sites were offered BMI screening regardless of risk, because there was no definitive way to identify those at highest risk for prediabetes, and targeting youth who appeared to be overweight would be inappropriate and could reinforce obesity-related stigma.

Adolescents referred by health care providers had higher rates of return for prediabetes screening. This finding may have been because of the greater perceived importance by patients of following up after their doctor’s recommendation, providers’ bias to refer patients they felt were more adherent, or the fact that youth who regularly see doctors may be more likely to come for other health testing. Of those tested, adolescents referred by clinicians also had higher rates of prediabetes than adolescents recruited in community sites. Although demographic characteristics and BMI did not differ by recruitment method, adolescents recruited from community sites had overall more healthful self-reported behaviors and attitudes than those recruited from clinical sites, which may account for the differences in prediabetes diagnosis rates. Other factors may account for the different rates of diagnosis, such as additional medical information available to clinicians that allowed them to select the highest-risk adolescents or additional differences in behaviors or related psychosocial or cognitive mediators.

We did not find significant differences by recruitment site in the number of adolescents with prediabetes enrolled in workshops, completing workshops, or attending follow-up visits. This finding implies that the participation of eligible youth in this type of research does not differ by the initial recruitment source.

Our study has limitations. This was a small pilot study conducted at a small number of sites in 1 urban community; because all participants were referred to the study, there may have been biases of selection and response (ie, social desirability). Still, this approach seems promising in recruiting vulnerable, hard-to-reach youth for research.

In summary, we worked with community and academic partners to develop a pilot community-based peer-led intervention to prevent adolescent diabetes. In this setting, 1 in 3 overweight or obese youth had undetected prediabetes. Community-based and clinical recruitment were both effective in diagnosing at-risk adolescents with prediabetes. Clinical sites yielded higher rates of diabetes risk on the basis of BMI and higher rates of return for screening and diagnosed prediabetes. However, the broad approach we used allowed access to more adolescents and opportunities for education about weight and diabetes in a community setting.
